# Supplementary material for: Comorbidities at Diagnosis, Survival, and Cause of Death in Patients with Chronic Lymphocytic Leukemia: A Population-Based Study
Source: Int J Environ Res Public Health. 2021 Jan 15;18(2):701. doi: 10.3390/ijerph18020701 (PMC7830671; doi:10.3390/ijerph18020701)
Supplement: Supplementary file 1 [file ijerph-18-00701-s001.zip › suppl/Supplementary Table 1.pdf]

Table S1. Specific cause of death by age group of patients with chronic lymphocytic leukemia in Girona, Spain.

Cause of death	CIE-10-ES code	Total	Age group		
			<65 years	65-78 years	>78 years
		n (%)	*n (%)*	*n (%)*	*n (%)*
**All**		**155 (100.0)**	**18 (11.6)**	**51 (32.9)**	**86 (55.5)**
**Hematological disease**		**86 (55.5)**	**10 (55.5)**	**29 (56.8)**	**47 (54.6)**
• Non-Hodgkin's lymphoma, unspecified	C859	8 (5.2)	-	4 (7.8)	4 (4.7)
• Chronic lymphocytic leukemia/small	C911	25 (16.1)	3 (16.7)	10 (19.6)	12 (14.0)
• Leukemia, unspecified	C959	4 (2.6)	-	-	4 (4.7)
• Other lymphoid leukemias	C917	35 (22.6)	4 (22.2)	9 (17.6)	22 (25.6)
• Lymphoid leukemia,unspecified	C919	3 (1.9)	-	2 (3.9)	1 (1.2)
• Acute leukemia, cells of unspecified type	C950	2 (1.3)	2 (11.1)	-	-
• Myelodysplastic syndrome, unspecified	D469	1 (0.6)	1 (5.6)	-	-
• Chronic leukemia of unspecified cell type	C951	2 (1.3)	-	1 (2.0)	1 (1.2)
• Myeloid Leukemia	C920	1 (0.6)	-	-	1 (1.2)
• Other myeloid leukemias	C927	1 (0.6)	-	1 (2.0)	-
• Small cell B-cell lymphoma	C830	2 (1.3)	-	1 (2.0)	1 (1.2)
• Unclassified peripheral T-cell lymphoma	C844	1 (0.6)	-	1 (2.0)	-
• Uncertain behavioral neoplasm of lymphatic, hematopoietic and related tissue, unspecified	D479	1 (0.6)	-	-	1 (1.2)
**Cardiovascular disease**		**11 (7.1)**	**1 (5.6)**	**1 (2.0)**	**9 (10.5)**
• Atrial fibrillation and flutter	I48	3 (1.9)	-	-	3 (3.5)
• Acute myocardial infarction, unspecified	I219	3 (1.9)	1 (5.6)	1 (2.0)	1 (1.2)
• Dilated myocardiopathy	I420	1 (0.6)	-	-	1 (1.2)
• Heart failure, unspecified	I509	1 (0.6)	-	-	1 (1.2)
• Left ventricular failure, unspecified	I501	1 (0.6)	-	-	1 (1.2)
• Chronic ischemic heart disease, unspecified	I259	1 (0.6)	-	-	1 (1.2)
• Other Hypertrophic Cardiomyopathies	I422	1 (0.6)	-	-	1 (1.2)
**Cerebrovascular disease**		**6 (3.9)**	**1 (5.6)**	**2 (3.9)**	**3 (3.5)**
• Cerebral infarction due to cerebral artery embolism	I634	1 (0.6)	1 (5.6)	-	-
• Cerebral infarction, unspecified	I639	3 (1.9)	-	-	3 (3.5)
• Other specified cerebrovascular diseases	I678	2 (1.3)	-	2 (3.9)	-
**Pulmonary disease**		**12 (7.7)**	**-**	**3 (5.9)**	**9 (10.5)**

• Chronic respiratory insufficiency	J961	1 (0.6)	-	-	1 (1.2)
• Unspecified respiratory failure	J969	1 (0.6)	-	-	1 (1.2)
• Chronic obstructive pulmonary disease with acute lower respiratory tract infection	J440	1 (0.6)	-	1 (2.0)	-
• Chronic obstructive pulmonary disease, unspecified	J449	3 (1.9)	-	2 (3.9)	1 (1.2)
• Diseases of the bronchi, not otherwise classified	J980	1 (0.6)	-	-	1 (1.2)
• Streptococcus pneumoniae pneumonia	J13	1 (0.6)	-	-	1 (1.2)
• Pneumonia, unspecified microorganism	J189	1 (0.6)	-	-	1 (1.2)
• Other lung disorders	J984	1 (0.6)	-	-	1 (1.2)
• Other specified respiratory disorders	J988	1 (0.6)	-	-	1 (1.2)
• Influenza due to other types of flu viruses identified with other respiratory manifestations	J101	1 (0.6)	-	-	1 (1.2)
**Renal disease**		**3 (1.9)**	**-**	**1 (2.0)**	**2 (2.3)**
• Hypertensive chronic kidney disease with stage 5 chronic kidney disease or end-stage renal disease	I120	1 (0.6)	-	-	1 (1.2)
• Acute renal failure, unspecified	N179	1 (0.6)	-	1 (2.0)	-
• Hypertensive chronic heart and kidney disease without heart failure	I131	1 (0.6)	-	-	1 (1.2)
**Metabolic disease**		**3 (1.9)**	**-**	**2 (3.9)**	**1 (1.2)**
• Diabetes Mellitus	E145	1 (0.6)	-	1 (2.0)	-
• Diabetes Mellitus unspecified	E149	1 (0.6)	-	1 (2.0)	-
• Chronic or unspecified duodenal ulcer with perforation	K265	1 (0.6)	-	-	1 (1.2)
**Cognitive disease**		**4 (2.6)**	**-**	**-**	**4 (4.6)**
• Unspecified dementia	F03	1 (0.6)	-	-	1 (1.2)
• Late-onset Alzheimer's disease	G301	1 (0.6)	-	-	1 (1.2)
• Alzheimer's disease, unspecified	G309	1 (0.6)	-	-	1 (1.2)
• Epilepsy, type not specified	G409	1 (0.6)	-	-	1 (1.2)
**Neoplasm**		**19 (12.3)**	**4 (22.2)**	**10 (19.6)**	**5 (5.8)**
• Lung	C349	4 (2.6)	-	3 (5.9)	1 (1.2)
• Colon	C189	3 (1.9)	-	3 (5.9)	-
• Lip, oral cavity and pharynx	D000	1 (0.6)	-	-	1 (1.2)
• Location not specified	C809	1 (0.6)	-	-	1 (1.2)
• Pancreas	C259	1 (0.6)	1 (5.6)	-	-
• Prostate	C61	1 (0.6)	1 (5.6)	-	-
• Kidney	C64	4 (2.6)	1 (5.6)	2 (3.9)	1 (1.2)
• Thorax	C493	1 (0.6)	-	1 (2.0)	-
• Uterus	C55	1 (0.6)	1 (5.6)	-	-

• Uterus (endometrium)	C541	1 (0.6)	-	1 (2.0)	-
• Bladder	C679	1 (0.6)	-	-	1 (1.2)
**Other causes**		**11 (7.1)**	**2 (11.1)**	**3 (5.9)**	**6 (7.0)**
• Liver abscess	K750	1 (0.6)	-	1 (2.0)	-
• Acute appendicitis	K359	1 (0.6)	1 (5.6)	-	-
• Atherosclerosis of native limb arteries	I702	1 (0.6)	-	-	1 (1.2)
• Bleeding from anus and rectum	K625	1 (0.6)	-	-	1 (1.2)
• Vascular bowel disorder, unspecified	K559	1 (0.6)	-	1 (2.0)	-
• Essential (primary) hypertension	I10	1 (0.6)	-	1 (2.0)	-
• Urinary tract infection, location not specified	N390	1 (0.6)	-	-	1 (1.2)
• Pathological fracture, not otherwise classified	M844	1 (0.6)	-	-	1 (1.2)
• Inhalation and ingestion of other objects that cause airway obstruction, in an unspecified location	W809	1 (0.6)	-	-	1 (1.2)
• Pedestrian injured in traffic accident with other motorized and unspecified vehicles	V092	1 (0.6)	1 (5.6)	-	-
• Poorly defined and unknown causes of mortality	R99	1 (0.6)	-	-	1 (1.2)

CIE-10-ES, Clasificación Internacional de Enfermedades-décima edición española.
