# Supplementary material for: Comorbidities at Diagnosis, Survival, and Cause of Death in Patients with Chronic Lymphocytic Leukemia: A Population-Based Study
Source: Int J Environ Res Public Health. 2021 Jan 15;18(2):701. doi: 10.3390/ijerph18020701 (PMC7830671; doi:10.3390/ijerph18020701)
Supplement: Supplementary file 1 [file ijerph-18-00701-s001.zip › suppl/Supplementary Table 2.pdf]

Table S2. Causes of death by reference characteristics in patients diagnosed with CLL.

Clinical features	Cause of death: Total *n* (%)[a]	CLL-related *n* (%)[a]	Unrelated to CLL *n* (%)[a]	p-value
**All**	**155**	**86 (55.5)**	**69 (44.5)**	
**Age**				
Mean (SD)	77.5 (10.1)	77.2 (10.3)	78.0 (10.0)	0.463
Median (Range)	79 (74-85)	79 (72.2-84.7)	80 (74-85)	
**Sex**				
Male	95 (61.3)	54 (62.8)	41 (59.4)	0.669
Female	60 (38.7)	32 (37.2)	28 (40.6)	
**Age group**				
<65	18 (11.6)	10 (11.6)	8 (11.6)	0.969
65-78	51 (32.9)	29 (33.7)	22 (31.9)	
>78	86 (55.5)	47 (54.7)	39 (56.5)	
**Rai stage**				
0	67 (43.2)	32 (37.2)	35 (50.7)	0.091
I-II	28 (18.1)	21 (24.4)	7 (10.1)	
III-IV	23 (14.8)	14 (16.3)	9 (13.0)	
Unknown	37 (23.9)	19 (22.1)	18 (26.1)	
**Period of diagnostic**				
2008-2010	76 (49.0)	46 (53.5)	30 (43.5)	0.327
2011-2013	58 (37.4)	31 (36.0)	27 (39.1)	
2014-2016	21 (13.5)	9 (10.5)	12 (17.4)	
**CCI score**				
0	2 (1.3)	1 (1.2)	1 (1.4)	0.243
1-2	8 (5.2)	7 (8.1)	1 (1.4)	
3-4	59 (38.1)	34 (39.5)	25 (36.2)	
>4	74 (47.7)	36 (41.9)	38 (55.1)	
Unknown	12 (7.7)	8 (9.3)	4 (5.8)	

a Except when specified; SD, standard deviation; CLL, Chronic lymphocytic leukemia; CCI, Charlson comorbidity index
